# Combination of Ginsenosides Rb2 and Rg3 Promotes Angiogenic Phenotype of Human Endothelial Cells via PI3K/Akt and MAPK/ERK Pathways

**DOI:** 10.3389/fphar.2021.618773

**Published:** 2021-02-10

**Authors:** Ran Joo Choi, Siti Zuraidah Mohamad Zobir, Ben Alexander-Dann, Nitin Sharma, Marcella K.L. Ma, Brian Y.H. Lam, Giles S.H. Yeo, Weidong Zhang, Tai-Ping Fan, Andreas Bender

**Affiliations:** ^1^ Department of Chemistry, Center for Molecular Science Informatics, University of Cambridge, Cambridge, United Kingdom; ^2^ Department of Pharmacology, University of Cambridge, Cambridge, United Kingdom; ^3^ Medical Research Council (MRC) Metabolic Diseases Unit, University of Cambridge Metabolic Research Laboratories, Wellcome–MRC Institute of Metabolic Science, Genomics and Transcriptomics Core, Addenbrooke’s Hospital, Cambridge, United Kingdom; ^4^ Department of Pharmacy, Second Military Medical University, Shanghai, China

**Keywords:** angiogenesis, tube formation, RNA-seq, weighted gene correlation network analysis, synergistic combination, shexiang baoxin pill, ginsenoside Rb2, ginsenoside Rg3

## Abstract

Shexiang Baoxin Pill (SBP) is an oral formulation of Chinese materia medica for the treatment of angina pectoris. It displays pleiotropic roles in protecting the cardiovascular system. However, the mode of action of SBP in promoting angiogenesis, and in particular the synergy between its constituents is currently not fully understood. The combination of ginsenosides Rb2 and Rg3 were studied in human umbilical vein endothelial cells (HUVECs) for their proangiogenic effects. To understand the mode of action of the combination in more mechanistic detail, RNA-Seq analysis was conducted, and differentially expressed genes (DEGs), pathway analysis and Weighted Gene Correlation Network Analysis (WGCNA) were applied to further identify important genes that a play pivotal role in the combination treatment. The effects of pathway-specific inhibitors were observed to provide further support for the hypothesized mode of action of the combination. Ginsenosides Rb2 and Rg3 synergistically promoted HUVEC proliferation and tube formation under defined culture conditions. Also, the combination of Rb2/Rg3 rescued cells from homocysteine-induced damage. mRNA expression of *CXCL8, CYR61, FGF16 and FGFRL1* was significantly elevated by the Rb2/Rg3 treatment, and representative signaling pathways induced by these genes were found. The increase of protein levels of phosphorylated-Akt and ERK42/44 by the Rb2/Rg3 combination supports the notion that it promotes endothelial cell proliferation *via* the PI3K/Akt and MAPK/ERK signaling pathways. The present study provides the hypothesis that SBP, *via* ginsenosides Rb2 and Rg3, involves the CXCR1/2 CXCL8 (IL8)-mediated PI3K/Akt and MAPK/ERK signaling pathways in achieving its proangiogenic effects.

## Introduction

Shexiang Baoxin Pill (SBP) is a traditional Chinese medicine (TCM) formulation taken orally to treat coronary heart disease (CHD) such as chronic stable angina (Lu et al., 2018). Like many other traditional medicines, SBP is derived from plant and animal products that contain a multitude of components which are thought to contribute to the holistic efficacy of the formulation (Yan et al., 2006; Yan et al., 2009). SBP has previously been shown to be protective against myocardial infarction (Wu et al., 2020) and associated oxidative injury and inflammation in rat models (Xiang et al., 2012; Xiang et al., 2013). Emerging pharmacological studies have revealed that SBP displays pleiotropic roles in protecting the cardiovascular system, as seen by the promotion of angiogenesis, amelioration of inflammation, improvement of endothelium dysfunction, mitigation of dyslipidemia, repression of vascular smooth muscle cell proliferation, and migration and restraint of cardiac remodeling (Lu et al., 2018).

On the mechanistic level, SBP has been shown to promote angiogenesis by stimulating proliferation, migration, and nitric oxide (NO) secretion by endothelial progenitor cells (Wu et al., 2009). According to a recent meta-analysis of 25 studies with 439 animals, SBP was shown to reduce myocardial infarction area through an increase of vascular endothelial growth factor (VEGF) and microvessel count in CHD, indicating that SBP’s cardioprotective effects are largely due to promotion of angiogenesis (Zhang et al., 2017). In a rabbit model of myocardial infarction, administration of SBP enhances angiogenesis in the infarcted area, whilst reducing capillary density at the atherosclerotic plaque site in animal models at the same time (Shen et al., 2010). In a previous study, one of us has shown that the mixture of several compounds can show much the same effects compared to the SBP extract by analyzing microarray data (Fang et al., 2017). In this study, we tested the hypothesis that a limited number of compounds found in SBP can synergistically interact with each other to play a major role in treating cardiovascular diseases by promoting angiogenesis. Therapeutic angiogenesis is a heavily investigated and promising method for treatment of refractory angina or myocardial ischemia, among other indications (Attanasio and Schaer, 2011; Mitsos et al., 2012).

In order to investigate the pro-angiogenic effect of SBP and to understand its mechanism of action further, suitable model systems are needed. Endothelial cells (ECs) are the principal players involved in angiogenesis, and their ability to divide and differentiate into tube-like structures *in vitro* has long been a strong indicator of their key role in physiological and pathological angiogenesis (Munoz-Chapuli et al., 2004) and thus, ECs can be used in an *in vitro* model to study angiogenesis. ECs are highly responsive to signals such as VEGF and fibroblast growth factors (FGFs) (Carmeliet and Jain, 2011). The pro-angiogenic response often involves multiple steps, including Akt activation (Fang et al., 2013) as well as activation of its downstream target, endothelial nitric oxide synthase (eNOS). NO release is one of the first events in the angiogenic cascade, preceding new lumen formation and even tip cell migration (Carmeliet and Jain, 2011). On the other hand, the mitogen-activated protein kinase (MAPK) pathway increases apoptotic pathway activated by environmental stressors that downregulates the pro-angiogenic VEGF pathway to mediate angiogenesis (Gomes and Rockwell, 2008), and accompanies serum-starved apoptosis in ECs (Yilmaz et al., 2003).

Human umbilical vein endothelial cells (HUVECs) are the most commonly used *in vitro* models of angiogenesis, where it is possible to observe their migration, proliferation and tubule formation in response to exogenous inhibitory or stimulatory agents (Staton et al., 2004). Overall, based on previous studies above, their frequent use as a model system, and their ability to recapitulate biologically relevant pathways (DeCicco-Skinner et al., 2014), we chose HUVECs in this work to untangle some aspects of the mode of action of angiogenesis modulators.

In a related study we modeled 22 chemicals identified in blood plasma after oral administration of SBP, and their ability in pairwise combinations to promote angiogenesis *in vitro* (Zobir et al., submitted). Here we systematically developed an algorithm to predict the synergy of all 231 pairwise combinations of those 22 compounds, which was also successfully experimentally validated. It was proposed from this previous work based on and *in silico* target prediction model that certain complementary pathways are responsible for the synergistic effect in promoting HUVEC proliferation. From the compound side, ginsenoside Rb2 in combination with ginsenoside Rg3 was found to dramatically increase proliferation of HUVECs, and protected these cells against homocysteine-induced damage. However, this previous study did not experimentally validate molecular mechanisms that caused those pro-angiogenic effects for this compound combination.

Hence, the present study aims to investigate the underlying mechanisms of the combination of ginsenosides Rg2 and Rg3 by utilizing RNA-sequencing (RNA-Seq) data generated in a biologically relevant system. The mRNA readout was analyzed by two standard methods in the field, namely Weighted Gene Co-expression Network Analysis (WGCNA) (Langfelder and Horvath, 2008) and Gene Set Enrichment Analysis (GSEA) (Subramanian et al., 2005), followed by expression validation using qPCR. In addition, we used pathway inhibitors to successfully verify the hypothesized modes of action, and applied phenotypic assays to further elucidate the mechanisms of this compound combination related to their modulation of angiogenic processes.

## Materials and Methods

### General Materials and Cell Culture

#### Ginsenosides

Ginsenoside Rb2 (purity >99.0%) was supplied by Shanghai Hutchison Pharmaceuticals Ltd. (Shanghai, China) (Fang et al., 2017), and ginsenoside Rg3 with >98.0% purity was supplied by Prof. Yeong Shik Kim, Seoul National University, Republic of Korea (Ha et al., 2007). Compounds were dissolved to 100 mM in DMSO, then subsequently diluted in media. The final concentration of DMSO was less than 0.1%, which is not toxic to cells, and the vehicle control also contained the same amount of DMSO as the compound solution to identify any solvent-induced effects.

#### Cells and Biological Tools

HUVECs and human dermal fibroblasts (HDFs) were purchased from PromoCell (Heidelberg, Germany). Cells were cultured in PromoCell’s Endothelial Cell Growth Medium 2 (EGM-2) containing 2% FBS, human epidermal growth factor (EGF), human basic fibroblast growth factor (FGF), insulin-like growth factor (IGF), human vascular endothelial growth factor 165 (VEGF_165_), ascorbic acid and heparin. Trypsin 0.005%/EDTA 0.01% solution, Dulbecco A phosphate buffered saline (PBS) and 1-Step™ NBT/BCIP (Pierce Protein Research) were obtained from Thermo Fisher Scientific (MA, U.S.A.). Fetal bovine serum (FBS), dimethyl sulfoxide (DMSO), paraformaldehyde, Triton^®^ X-100, rabbit anti-human von Willebrand factor antibody (F-3520), and mouse anti-rabbit IgG-alkaline phosphatase (A9919) were purchased from Sigma-Aldrich (Gillingham, United Kingdom). Human recombinant VEGF and precast gels were obtained from Invitrogen Life Technologies (Paisley, United Kingdom) and antibodies for Western blotting were sourced from Cell Signaling through New England Biolabs (Hitchin, United Kingdom).

### Cell Viability Measurement

The effect of compounds and compound combinations on cell proliferation *in vitro* was assessed by the Cell Counting Kit-8 (CCK-8) assay (Dojindo, Kumamoto, Japan). CCK-8 utilizes the highly water-soluble tetrazolium salt WST-8, and dehydrogenases within living cells reduce WST-8 to produce colored (orange) formazan. The amount of formazan dye generated is then proportional to the number of living cells.

HUVECs were seeded onto 96-well flat-bottom plates at a density of 2.5 × 10^3^ cells per well in 100 µl of EGM-2. Following incubation for 24 h, the culture medium was aspirated and replaced with diluted drug solutions or vehicle controls in Endothelial Cell Basal Medium 2 (EBM-2, PromoCell). Combinations of ginsenoside Rb2 (10, 50, 100 µM) and ginsenoside Rg3 (1, 5, 10 µM) were treated in a 4 × 4 checkerboard arrangement allowing synergy to be quantified, and therefore the best pair of concentrations of each compound can be determined (Hsieh et al., 1993; Orhan et al., 2005; Vlot et al., 2019). After incubation for a further 72 h, 10 μL of CCK-8 (Dijindo) was added to each well, and plates were incubated for 2 h in darkness at 37°C. Absorbance at 450 nm was then measured with a MultiSkan Ascent Plate Reader (MTX LAB SYSTEMS, Florida, U.S.A).

For sulforhodamine B (SRB) assay, HUVECs were treated with drug solutions or vehicle controls under the same culture condition as above. After 72 h of incubation, media was removed and then cells were fixed in cold 3% Trichloroacetic acid (TCA) for 30 min at 4°C. 0.057% SRB in 1% acetic acid were added to the cells and images were captured. The ImageJ software was used to count cell numbers present in each image.

### Bliss Independence Model Measurement

The combination response (4 × 4) matrix contained percentages of cell growth, with cell growth without compound treatment set to 100%. Since the compounds applied either individually or in combination are assumed to promote cell growth, each value in the matrix was subtracted from 100 to produce percentage cell growth starting from 0%. A single-compound dose response curve was fitted to the data using the 4-parameter log-logistic function (4-PL) with the Synergy Finder package in R (R Development Core Team, 2010; He et al., 2018). Subsequently, the synergy of the combination was calculated using the Bliss model (Bliss, 1939), which assumes the drugs to act *via* independent mechanisms (For a discussion of the theoretical background and advantages and disadvantages of the different synergy models see a recent review (Vlot et al., 2019)). The Bliss score of the combination was calculated by averaging the difference between the actual percentage of cell growth and the expected percentage of cell growth with four replicate values, also in Synergy Finder.

### IncuCyte Time-Lapse Imaging

Images were acquired with the IncuCyte Live Cell Imaging microscopy (Essen Bioscience) every 3 h up to 72 h under cell culture conditions with a 10X objective. Averaged cell confluence was calculated from three random fields of view per well using the IncuCyte in-built algorithm. Relative confluence values were obtained by normalizing each value to the time zero value in each sample.

### Homocysteine-Induced Damage in a Co-culture Tube Formation Assay

The tube formation assay used here is based on the Bishop Model of co-culture tube formation (Bishop et al., 1999). Briefly, HUVECs and HDFs were cultured in EGM-2 until sub-confluent. Cells were then seeded in 48-well flat-bottom plates at a 1:20 ratio at around 2,000 HUVECs to 40,000 HDFs per well in EGM-2 medium. After two days, media were replenished, and on day 4, to allow for detection of protective effects of drugs against homocysteine (HCY)-induced damage, EGM-2 was titrated 10-fold with its basal medium (EBM-2) and 2% FBS. After 4 h of incubation with ginsenoside Rb2 (50 µM) alone, ginsenoside Rg3 (5 µM) alone, or the Rb2/Rg3 combination showing the highest Biliss synergy score, 2 mM HCY was added. These reagents in fresh media were replenished every two days before staining on day 12.

For staining, cells were first fixed and permeabilized with 4% paraformaldehyde in PBS, and an anti-human-vWF monoclonal antibody (dilution ratio 1:1,000; Sigma-Aldrich, MO, U.S.A.) was added (dilution ration 1:1,000) for 1 h before staining with an anti-rabbit alkaline phosphatase conjugated antibody (dilution ratio 1:1,000) for 45 min. The 1-Step™ NBT/BCIP (Thermo Fisher Scientific, MA, U.S.A) was applied until a suitable signal was developed (10–15 min). Images of two fields of view were taken for three wells each, and total tube area measured using the ImageJ software. Total tube area was calculated as the number of connected pixels using “Analyze particles” above a certain size threshold (200-∞) following a binary normalization with an automatic threshold level.

### RNA-Sequencing

Total RNA was isolated from cells using the RNeasy Kit (Qiagen) according to the manufacturer’s protocol. An additional DNase1 digestion step was performed to ensure that the samples were not contaminated with genomic DNA. One microgram of total RNA, after validation by an Agilent Bioanalyser 2,100 system to have an appropriate RIN between 7.6–10, was used for library construction using Illumina's TruSeq Stranded mRNA Library Prep Kit (Illumina, CA, U.S.A) at the Institute of Metabolic Science Genomics and Transcriptomics Core Facility (Cambridge, United Kingdom). Messenger RNA was enriched by poly-T oligo attached magnetic beads before reverse transcription. Addition of a single “A” nucleotide (adenylation) after the synthesis of the double-stranded cDNA stopped the ligation of DNA fragments during the adapter ligation reaction. Unique barcodes were added to individual samples allowing multiplexed sequencing. DNA fragments with successful adapters ligated were enriched with a limited amplification. The second strand cDNA, with the incorporation of dUTP, was quenched during amplification to allow strand-specific sequencing. Indexed libraries were purified, normalized, pooled and sequenced on the Illumina HiSeq 4,000 platform at single read length of 50 base pairs at the Genomics Core Facility, Cancer Research United Kingdom Cambridge Institute (Cambridge, United Kingdom). The raw count data can be found at the following link. https://www.ncbi.nlm.nih.gov/Traces/study/?acc=PRJNA671806.

### Analysis of Differentially Expressed Genes

The quality of the reads was examined using FastQC (Andrews, 2010) and Trimmomatic (Bolger et al., 2014). An index for the reference genome, GRCh38 (Kersey et al., 2016) was created using Bowtie2 (Langmead and Salzberg, 2012). Reads were then mapped to the reference genome using Tophat2 (Kim et al., 2013) with the --b2-very-sensitive option. HTSeq-0.6 (Anders et al., 2015) was used to count the number of reads sorted by read name using SAMtools (Li et al., 2009). Differentially expressed genes (DEGs) between the untreated control and compound-treated groups were estimated using the DESeq2 statistical package (Love et al., 2014) with significant genes defined as those with a False Discovery Rate (FDR) ≤0.01.

### Weighted Correlation Network Analysis

The reads were matched to genes using the AnnotationDbi (Pagès et al., 2018) and org.Hs.eg.db (Carlson, 2019) packages in R (version 3.5.1) (R Core Team, 2018). Genes with zero variance across all samples (control as well as Rb2, Rg3, Rb2+Rg3 and FGF-treated) were removed. The remaining samples were hierarchically clustered using average linkage and Euclidean distance across all genes in R using the “wgcna” package (Langfelder and Horvath, 2008). The unsigned network was built using correlation of genes with the parameters softpower β = 10, mergeCutHeight = 0.15, detectCutHeight = 0.995, and maxBlockHeight = 32,000. Module size has a positive correlation with Zsummary, a score that determines module preservation (Langfelder et al., 2011). Choosing a larger number of similarly sized modules allows for a fair comparison of the modules, and hence was used for subsequent analysis. A Fisher’s Exact Test was performed in WGCNA package to measure the categorical overlap between the resultant modules. Module preservation and reproducibility were determined using a permutation of the inner- and intra-connectivity, eigengenes and densities between the compound induced and control networks (Langfelder et al., 2011), resulting in Zsummary scores and median ranks. A previously suggested cutoff (Langfelder et al., 2011) of Zsummary scores >10 for strong evidence of conservation was applied, and 15 modules were excluded as these were not significantly conserved when compared to the control group (and so are assumed to represent unperturbed biological mechanisms). Genes were filtered for intramodular correlation (>0.7) and significance (<0.01) to focus on genes that are central to the modules, in the connectivity space. In this manner, the remaining genes are significantly correlated to the module, and so increases the likelihood that they are assigned to the correct module.

### Gene Set and Pathway Analysis

Gene sets were analyzed using DAVID v 6.8 (Huang et al., 2009b; a) to determine enriched Gene Ontology (GO) Biological Processes of differentially expressed genes in the combination-treated group, compared to control and highly enriched pathways (*p* value <0.05) were identified with their constituent genes. DAVID analysis was conducted under medium stringency for biological process enrichment (Category: GOTERM_BP_DIRECT) and annotation clusters were summarized manually for representation purposes. To detect overrepresentation of genes in canonical pathways, differentially expressed genes were compared to the background of all measure genes. Genesets used were the Molecular Signatures database v 6.1 (Subramanian et al., 2005) (http://www.broadinstitute.org/gsea/msigdb/index.jsp), and overlap of genes in a pathway level interaction network was conducted with the ConsensusPathDB release 30 (January 09, 2015).

### Quantitative Real Time PCR

A manually selected subset of genes of particular interest originating from the above analyses (pathway annotation and WGCNA analysis of RNA-seq results) were validated experimentally through quantitative real-time polymerase chain reaction (qPCR). Specific primers for selected genes were designed according to literature (Supplementary Table S2). All samples were amplified in triplicates and RNA utilized for real-time qPCR was extracted with RNeasy as described above. Purified RNA was reverse transcribed using the iScript™ cDNA Synthesis Kit (BIO-RAD) following the manufacturer protocol. Real time PCR amplifications were performed in Quantstudio 5 (Thermo Fisher Scientific, MA, U.S.A.) with an initial hot start of 95°C for 15 min followed by 45 cycles of 95°C for 15 s and 60°C for 30 s. In order to normalize qPCR reactions, GAPDH (Glyceraldehyde 3-phosphate dehydrogenase) was included as housekeeping gene. The relative expression of genes was calculated based on the average Ct values across samples and significance was determined using the Students *t*-test (Microsoft Excel 2019, Microsoft, WA, U.S.A.), comparing control vs. the combination-treated mean values.

### Western Blotting

Western blots were performed on HUVECs grown in 6-well plates. Following 30 min of incubation with drug or vehicle total cell protein was harvested using a lysis buffer (50 mM Tris-HCl, pH 7.5, 250 mM NaCl, 5 mM EDTA, 50 mM sodium fluoride, 0.5% sodium deoxycholate, 0.5% Triton X-100, protease inhibitor cocktail) and protein concentration was measured using the BCA Protein Assay Kit (Pierce Protein Research, Thermo Fisher Scientific, MA, U.S.A.). Equal amounts of protein were loaded into a 4–12% Bis-Tris gel (Invitrogen, Paisley United Kingdom) before being transferred onto a nitrocellulose membrane (Thermo Fisher Scientific, MA, U.S.A.). The membranes were blocked in TBS-T + 5% milk and then stained using primary antibodies (dilution ratio 1:1,000) overnight at 4°C, or at room temperature for 1 h with gentle shaking in TBS-T solution. Following three washes, horseradish peroxidase-conjugated secondary antibody (rabbit anti-mouse, 1:5,000) was added for 1 h at room temperature with gentle shaking. The signal was developed using Amersham ECL Prime (GE Healthcare, Buckinghamshire United Kingdom).

### Statistical Analysis

An ordinary one-way ANOVA with a Tukey’s multiple comparisons test or a non-parametric Kruskal–Wallis one-way analysis was performed on cell-based *in vitro* data using GraphPad Prism version five for Windows (GraphPad Software, CA, USA, www.graphpad.com), where the mean of each treatment group was compared with the mean of the control. *p* < 0.05 were considered as statistically significant.

## Results

### Synergistic Effects of Ginsenosides Rb2 and Rg3 on Promoting Endothelial Cell Proliferation

**FIGURE 1:**
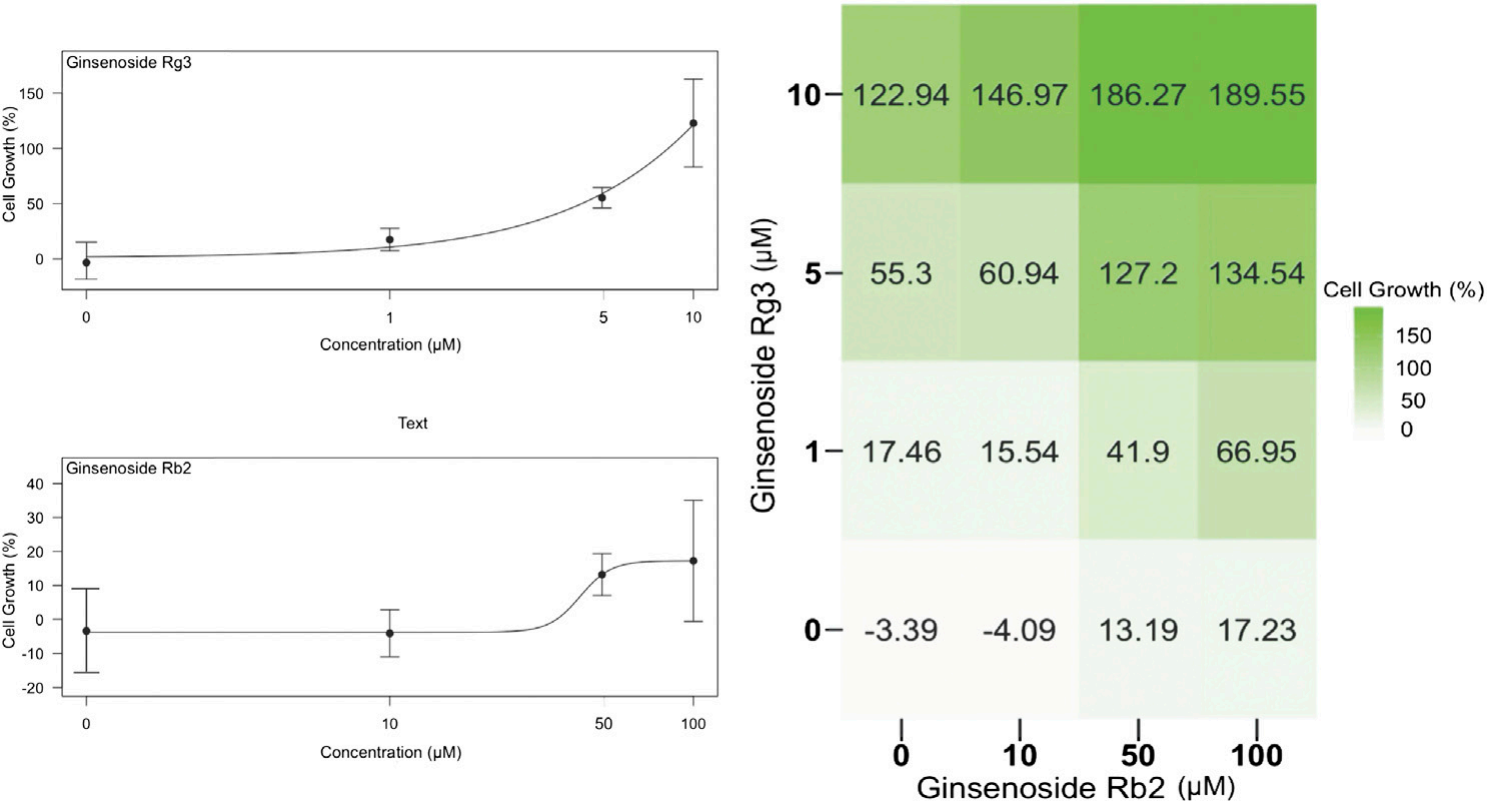
Effect of individual ginsenosides Rg3 and Rb2 on cell growth on a semi-logarithmic plot **(left)** and the 4 × 4 combination response matrix **(right)**. By calculating the synergy score with the Bliss method, ginsenoside Rb2 and ginsenoside Rg3 synergistically promoted endothelial cell growth with a Bliss synergy score of 39.48. Data are represented as mean ± SD, n = 4.

We first sought to characterize the effects of each single ginsenoside on HUVEC proliferation to determine the optimal concentration range for experiments. Since ginsenoside Rg3 is known to show cell cytotoxicity at higher doses and thus widely used in cancer treatments (Joo et al., 2015), we decided to use concentrations of 10–100 µM for ginsenoside Rb2 and 1–10 µM for ginsenoside Rg3 individually as well as in a 4 × 4 concentration matrix (Figure 1). On the left side, individual effects of ginsenoside Rb2 or ginsenoside Rg3 on HUVEC proliferation were shown. Both compounds have enhancing effects over the basal growth, notably ginsenoside Rg3 promotes cell proliferation from 20% at 1 μM to 120% at 10 μM, whereas ginsenoside Rb2 produced a weaker proliferating effect, showing a sigmoidal curve. On the right side of Figure 1, the percentage of the increase in cell proliferation can be seen in a checkerboard format with concentrations of each compound on *X* and *Y* axis. A Bliss (Bliss, 1939) synergy score of 39.48 was obtained in combination-treated cells (Figure 1) which strongly supported that ginsenoside Rb2 and ginsenoside Rg3 synergistically promote endothelial cell proliferation. A Bliss synergy score above 0 indicates synergism throughout the pairwise concentrations tested, thus 39.48 provides strong support to suggest that ginsenoside Rb2 and ginsenoside Rg3 can be a good combination in terms of promoting endothelial cell proliferation.

**FIGURE 2:**
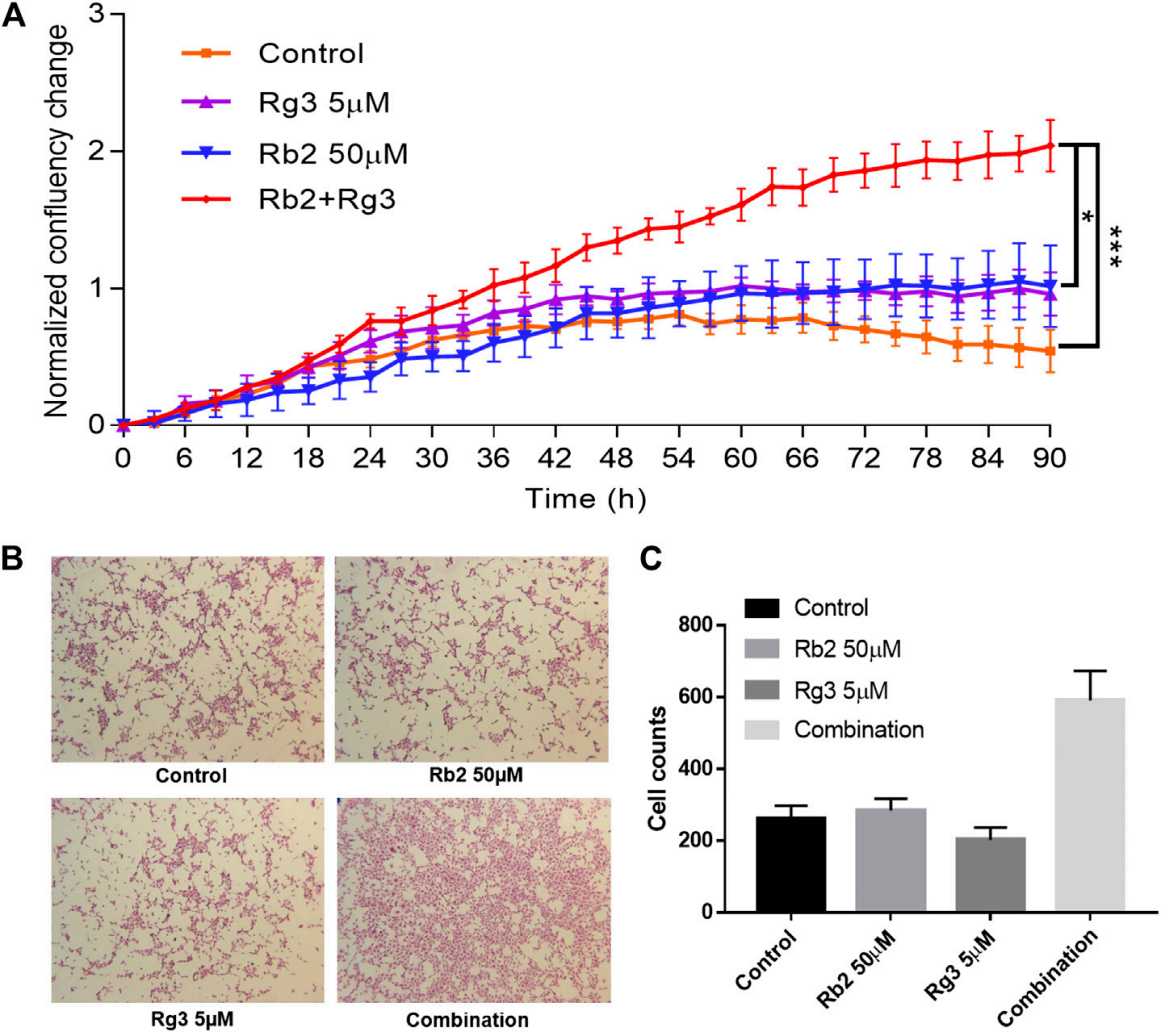
Effect of ginsenosides Rg3 and Rb2 on HUVEC proliferation as monitored by confluence of cell images every 3 h up to 90 h **(A)**. The mean fold change in confluence from the vehicle or compound alone or combination-treated group is shown. Confluence is a suitable surrogate for proliferation and cell growth over time, until the point that cells are densely packed. It can be seen that the combination of these ginsenosides approximately doubled the cell growth rate over individual treatments, and even more over control (no treatment). The representative images of SRB stained cells were shown **(B)**. The ImageJ software was used to count the number of stained cells and as a result the combination significantly increased cell counts **(C)**. Data represents mean ± SD, n = 4. * indicates *p* < 0.05 and *** indicates significance at *p* < 0.001 (based on the Kruskal-Wallis non-parametric test).

To investigate the time course of the synergistic effects of the combination of ginsenosides Rb2 (50 µM) and Rg3 (5 µM) on HUVEC growth, the IncuCyte system was used to track confluency changes. Since drugs were dissolved in medium without growth factors and nutrients the cell growth rate in the control wells was slightly decreased over time (Figure 2A). On the other hand, it was found that the cell population was increased 2-fold at 72 h compared to time zero by the combination of both ginsenosides, compared to either single agent or vehicle control (with *p* < 0.001, Figure 2). The results show that the combination of ginsenosides Rb2 and Rg3 can act like a replacement for essential growth supplements to promote cell proliferation when HUVECs are starved.

To add more convincing results, the SRB assay data were included in Figure 2. Representative images of stained cells after washout of floating cells in each group were shown in Figure 2B. Notably, the combination of Rb2 50 µM and Rg3 5 µM significantly promoted HUVEC cell growth (Figure 2C). The CCK-8 assay, the IncuCyte Live Cell Imaging and the SRB array differ in how they count the cell number, therefore the readouts of each method can vary. From the collection of obtained results, it is confirmed that the combination showed ability to increase cell growth compared to either the vehicle control or the single treated group.

### Ginsenosides Rb2 and Rg3 Protect Human Umbilical Vein Endothelial Cells Against Homocysteine-Induced Damage in a Co-culture Tube Formation Model

**FIGURE 3:**
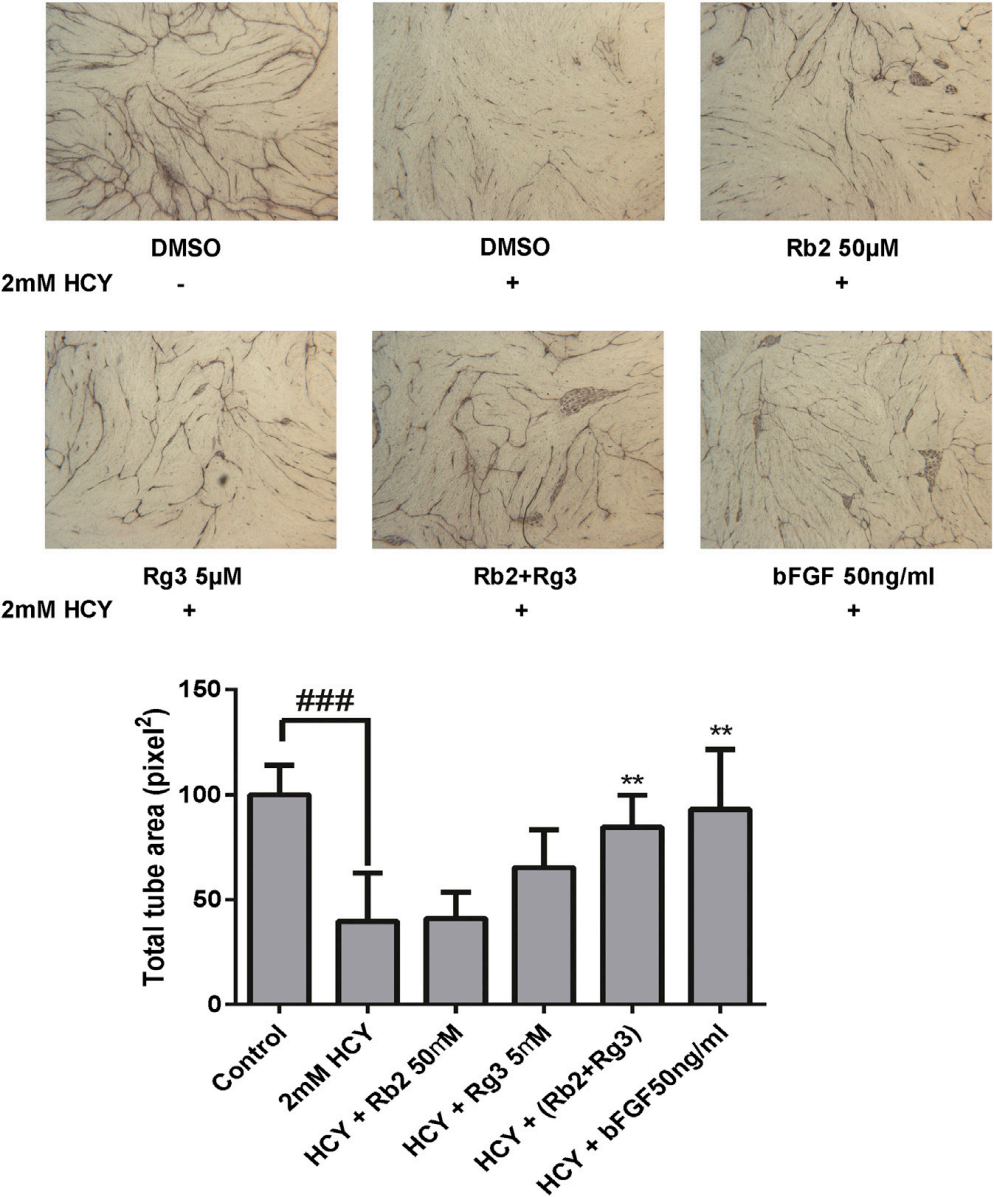
The combination of ginsenosides Rb2 and Rg3 protected endothelial cell tube against homocysteine (HCY)-induced damage in a co-culture tube formation model. The protective effect of the ginsenoside Rb2/Rg3 combination was as good as the positive control FGF. Data represents mean ± SD (n = 3). ### indicates *p* < 0.001; ** indicates *p* < 0.01 for treatment rescue compared to homocysteine-induced damage (based on tukey’s one-way ANOVA).

We next investigated the ability of endothelial cells to remodel and align *via* an *in vitro* tube formation assay, which is a requirement for the formation of new blood vessels during angiogenesis (Bishop et al., 1999). This method follows the interactions between endothelial cells and fibroblasts over 12 days leading to formation of vascular tubes with lumen and varying lengths, and is hence considered a better proxy (Gagnon et al., 2002) for the *in vivo* situation than the 6-h Matrigel method (Staton et al., 2004). Adding HCY to this co-culture tube formation model enables us to predict the ability of drugs to protect blood vessels against damage elicited by hyperhomocysteinemia. In the vehicle control group, co-culture of HUVECs and fibroblasts successfully formed vascular tubes which look like black thread, whilst 2 mM HCY destructed them. However, when the positive control FGF was treated before HCY induction, tube-like structures were still formed, indicating FGF had ability to protect HCY-induced vascular damage (Figure 3). Likewise, the combination of 50 μM ginsenoside Rb2 and 5 μM ginsenoside Rg3 (with highest Bliss synergy) clearly protected HUVEC tube-like structures against 2 mM HCY-induced damage and hence reversed the damaged phenotype. Quantitative measure of areas of tube-like structures was conducted to confirm the protective effect of the combination and presented as a bar graph in Figure 3.

### Distinct Gene Expression Patterns of Human Umbilical Vein Endothelial Cells Treated With Ginsenosides Rb2 and Rg3

We next analyzed the gene expression profile of HUVECs treated with either ginsenoside Rb2 (50 µM), ginsenoside Rg3 (5 µM) or Rb2/Rg3 combination in comparison to vehicle control in order to elucidate the mechanistic basis of compound synergy. This analysis resulted in 377 and 45 significantly differently expressed genes (DEGs; FDR <0.01) in the combination-treated group and the ginsenoside Rb2-treated group, respectively, compared to control, while no significant DEGs was observed on treatment of Rg3. Hence, on the overall level, when ginsenosides Rb2 and Rg3 are applied jointly, many more genes are altered than the sum of genes perturbed by the individual ginsenoside treatments, indicating compound interactions beyond simple additivity.

The 377 DEGs in the combination-treated cells were next subjected to Gene Set Enrichment Analysis (Subramanian et al., 2005), and the resulting ten highly enriched pathways with lowest *p* value are presented in Table 1. Extracellular matrix-related pathways are seen to be differentially regulated, indicating the pro-angiogenic effects by ginsenoside Rb2/Rg3 are at least partially due to extracellular matrix modulation (Sottile, 2004).

**TABLE 1:** Canonical pathways differentially expressed by Rb2/Rg3 combination treatment according to GSEA analysis (10 pathways with lowest *p*-value are shown).

Gene Set Name	# Genes in Gene Set (K)^1^	Description	# Genes in Overlap (k)^2^	k/K^3^	*p*-value	FDR q-value
NABA_MATRISOME	1,028	Ensemble of genes encoding extracellular matrix and extracellular matrix-associated proteins	49	0.0477	2.42 × 10^−24^	3.22 × 10^−21^
REACTOME_HEMOSTASIS	466	Genes involved in hemostasis	26	0.0558	5.76 × 10^−15^	3.65 × 10^−12^
NABA_MATRISOME_ASSOCIATED	753	Ensemble of genes encoding ECM-associated proteins including ECM-affiliated proteins, ECM regulators and secreted factors	32	0.0425	8.24 × 10^−15^	3.65 × 10^−12^
KEGG_FOCAL_ADHESION	201	Focal adhesion	17	0.0846	4.27 × 10^−13^	1.42 × 10^−10^
PID_FRA_PATHWAY	37	Validated transcriptional targets of AP1 family members Fra1 and Fra2	9	0.2432	8.65 × 10^−12^	2.30 × 10^−9^
NABA_SECRETED_FACTORS	344	Genes encoding secreted soluble factors	19	0.0552	3.32 × 10^−11^	7.36 × 10^−9^
KEGG_CYTOKINE_CYTOKINE_RECEPTOR_INTERACTION	267	Cytokine-cytokine receptor interaction	17	0.0637	4.03 × 10^−11^	7.65 × 10^−9^
NABA_CORE_MATRISOME	275	Ensemble of genes encoding core extracellular matrix including ECM glycoproteins, collagens and proteoglycans	17	0.0618	6.39 × 10^−11^	1.06 × 10^−8^
KEGG_PATHWAYS_IN_CANCER	328	Pathways in cancer	18	0.0549	1.22 × 10^−10^	1.81 × 10^−8^
PID_INTEGRIN3_PATHWAY	43	Beta3 integrin cell surface interactions	8	0.186	1.28 × 10^−9^	1.71 × 10^−7^

^1^ total number of genes in each gene set (K)

^2^ number of genes of our DEGs in each gene set (k)

^3^ ratio of k to K

**FIGURE 4:**
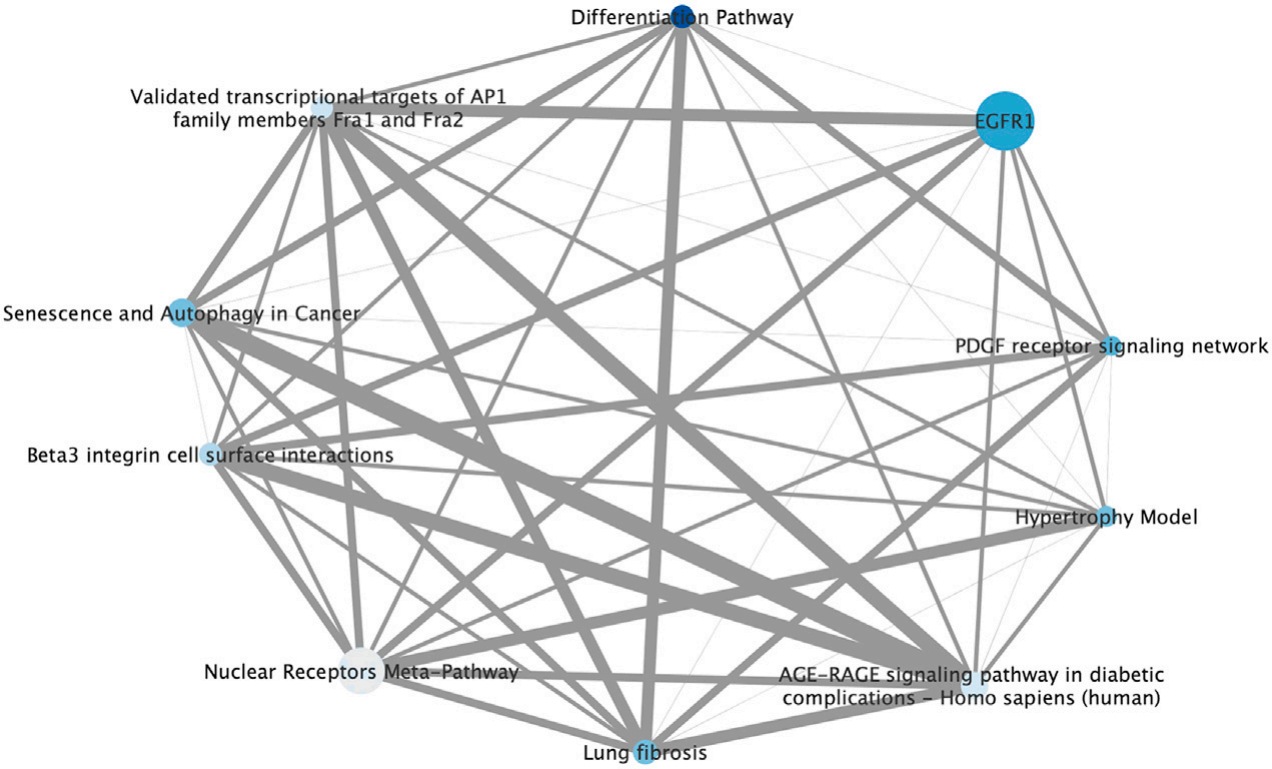
Pathway level interaction network of 10 highly enriched pathways in the ginsenoside Rb2/Rg3 combination-treated cells annotated according to ConsensusPathDB compared to control, providing synergy hypotheses of the compound combination. Node size is based on number of genes represented in each pathway gene set, color is determined by statistical enrichment value (darker blue = higher *p* value) and edge thickness represents the total number of genes shared between each pathway. (Detailed information about each pathway and its *p* value can be seen in Table 2.) It can be seen that growth factor receptor-mediated pathways such as EGFR1 and PDGF are major signaling that may contribute promoting effects on endothelial cell proliferation. Also, other pathways which are AP1, autophagy differentiation pathways are also known to be involved in regulating cell growth.

Next, we conducted Consensus PathDB (Kamburov et al., 2013) for the 377 DEGs to obtain an alternative view on this information, such as interactions between pathways. The 10 most highly enriched pathways are listed in Table 2 and visualized as a pathway level interaction network in Figure 4. Highly enriched pathways can be seen to be associated with cell growth and cell differentiation such as the EGFR, PDGFR and AP1 pathways, which hence form the mode of action hypothesis for angiogenesis modulation based on this part of the analysis. Ginsenoside Rb2 has been reported to promote epidermal proliferation via enhancing EGFR-related growth factors (Choi, 2002). Given that EGFR and PDGF are known receptors to promote angiogenesis, and AP1 families are important transcription factors to increase cell proliferation, we expect that the combination of ginsenoside Rb2 and Rg3 may target one or more of these pathways to produce overall effects.

**TABLE 2:** Overrepresentation analysis using Consensus PathDB comparing differentially expressed genes in ginsenoside Rb2/Rg3 combination treated cells to control (10 pathways with lowest *p*-value are shown). Growth factor-involved signaling pathways, such as PDGF, EGFR1 and differentiation pathway, are overrepresented as well as cell survival pathways such as AP1, senescence and autophagy in cancer.

*p*-value	q-value	Pathway	Source
1.44 × 10^−7^	0.000149	Validated transcriptional targets of AP1 family members Fra1 and Fra2	Pathway interaction database (PID) Schaefer et al. (2009)
8.07 × 10^−7^	0.000417	Senescence and autophagy in cancer	Wikipathways Kelder et al. (2009)
3.34 × 10^−6^	0.000856	AGE-RAGE signaling pathway in diabetic complications - homo sapiens (human)	KEGG Kanehisa and Goto (2000)
3.96 × 10^−6^	0.000856	PDGF receptor signaling network	PID
4.15 × 10^−6^	0.000856	Hypertrophy model	Wikipathways
5.11 × 10^−6^	0.000879	Beta3 integrin cell surface interactions	PID
6.06 × 10^−6^	0.000893	EGFR1	NetPath Kandasamy et al. (2010)
1.20 × 10^−5^	0.00143	Differentiation pathway	Wikipathways
1.25 × 10^−5^	0.00143	Nuclear receptors meta-pathway	Wikipathways
1.44 × 10^−5^	0.00143	Lung fibrosis	Wikipathways

### Weighted Gene Correlation Network Analysis

Initially, all cases datasets–FGF treated positive control, individual Rb2 and Rg3 treatments, Rb2/Rg3 combination treatment and vehicle control - were hierarchically clustered in gene expression space (shown in Supplementary Figure S1). This separated FGF-induced cells from the others, suggesting a separate mode-of-action, which was to be expected as a positive control.

We next applied WGCNA to create modules of co-expressed genes for the combination-treated group and tested these modules based on their conservation against control networks, the results of which are shown in Supplementary Table S1. 78 modules were found to not be conserved against control. Networks were created for each condition and modules formed at four levels, with each level representing cutting the tree at different heights (Supplementary Figure S2). Level 4, the lowest cut level, contained the large number (93) of distinct and specific modules compared to the other levels (which provided 56, 52, and 38 modules, respectively). The white module was both perturbed (i.e. not conserved) and contained the largest number of DEGs (Supplementary Figure S3). The combination of genes that are both differentially expressed and that are co-expressed (in the same module) could suggest the genes mutual involvement in the mode-of-action. Hence the white module was selected for further study.

**FIGURE 5:**
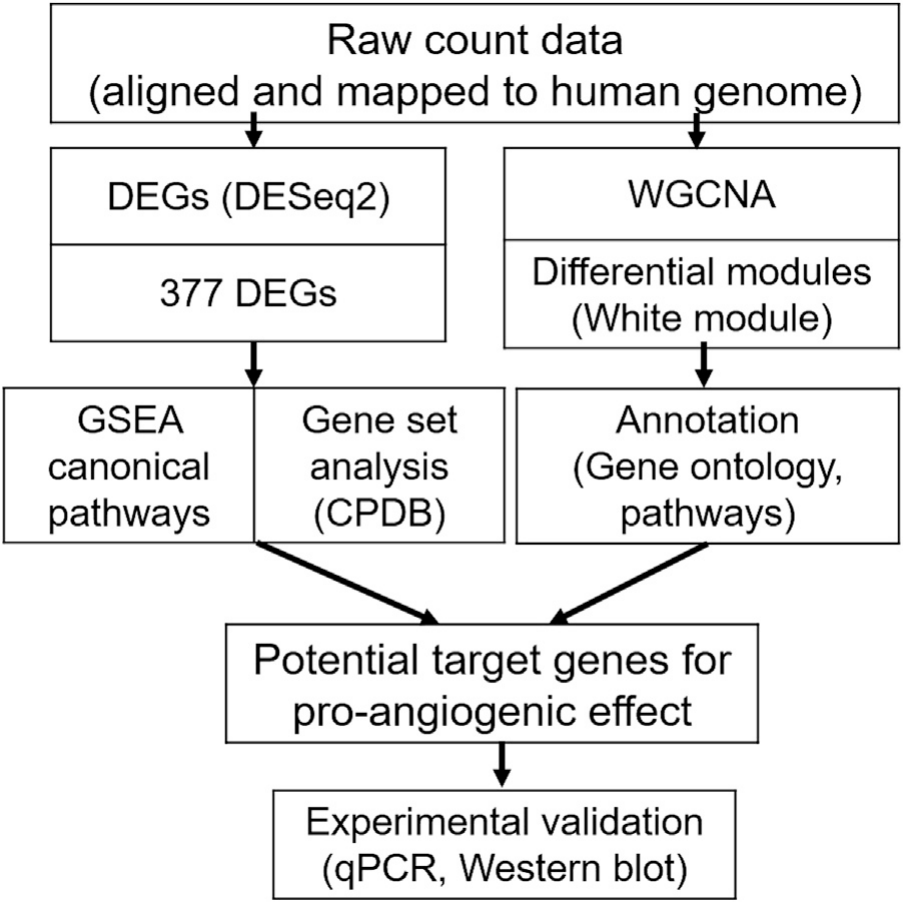
Workflow of the RNA-sequencing (RNA-Seq) analysis for the ginsenoside Rb2/Rg3 combination, compared to the control group. Firstly **(left)**, differentially expressed genes were determined using DESeq2. The 377 differentially expressed genes in the combination-treated group were subject to Gene Set Enrichment Analysis (GSEA) and Consensus Path DB (CPDB) gene set analyses. In parallel **(right)**, Weighted Gene Co-expression Network Analysis (WGCNA) was performed on combination treated vs untreated cells, and the resulting white gene module was subject to Gene Ontology and pathway analysis. Both methods together were used to understand the synergistic effect of the ginsenoside combination treatment on a mechanistic level, and formed the basis of selecting 18 genes for further experimental validation by qPCR and Western blotting.

All modules were then annotated with GO terms and, for example, the white module was found to have “angiogenesis (GO:0001525)” among its significant terms (Table 3). As shown in Figure 5, we conducted DESeq2 and WGCNA in parallel using RNA-seq data. By WGCNA, we found modules that shared no co-expression networks between control and treated groups and created 96 modules (deep split3) for the control and the combination groups. As a result, the white module showed the most significantly different co-expression between two groups (Seumois et al., 2016). In Table 4, the first six genes were selected because they were suggested by the WGCNA method with GO annotation (White GO module), while the remaining 12 genes were identified by DESeq2 in combination with either GSEA or CPDB pathway analysis.

**TABLE 3:** Gene Ontology analysis of Biological Processes of 94 genes found in the White Module, which is not conserved with the control network and contains the highest number of differentially expressed genes that correlated highly with the modules. It shows that this module is enriched in cell survival and angiogenesis signaling pathways, and hence these genes are of significant interest in determining the combination mode of action.

Term	Description	*p*-value
GO:0097191	Extrinsic apoptotic signaling pathway	0.0147
GO:0001525	Angiogenesis	0.0169
GO:0090050	Positive regulation of cell migration involved in sprouting angiogenesis	0.0304
GO:0008152	Metabolic process	0.0380
GO:0097105	Presynaptic membrane assembly	0.0390
GO:0070886	Positive regulation of calcineurin-NFAT signaling cascade	0.0390
GO:0043085	Positive regulation of catalytic activity	0.0496

**TABLE 4:** List of 18 genes selected from preceding analyses for confirmation by qPCR. The first six genes were suggested by the WGCNA method with GO annotation (White GO module), while the remaining 12 genes were identified by DESeq2 in combination with either GSEA or CPDB pathway analysis. Five genes (CIB1, MMP2, TNFRSF12A, FGF16, FGFRL1) have been chosen by the WGCNA method only and not by DESeq2, indicating those genes from the most highly correlated module may still be important in angiogenesis pathways, despite not being significantly differentially expressed.

Gene symbol	Gene name	Module	Evidence
CIB1	Calcium and integrin binding 1	White	White GO
MMP2	Matrix metallopeptidase 2	White	White GO
SERPINE1	Serpin family E member 1	White	White GO/GSEA/CPDB
TNFRSF12A	TNF receptor superfamily member 12A	White	White GO
FGF16	Fibroblast growth factor 16	White	White GO
FGFRL1	Fibroblast growth factor receptor-like 1	White	White GO
SPARC	Secreted protein acidic and cysteine rich	Purple	GSEA
PDGFA	Platelet derived growth factor subunit A	palevioletred3	GSEA/CPDB
SMOC1	SPARC related modular calcium binding 1	palevioletred2	GSEA
GDF3	Growth differentiation factor 3	navajowhite2	GSEA
TGFBR2	Transforming growth factor beta receptor 2	Lightyellow	GSEA/CPDB
PDGFRB	Platelet derived growth factor receptor beta	Darkmagenta	GSEA/CPDB
CYR61	Cysteine rich angiogenic inducer 61	Darkgrey	GSEA/CPDB
ITGB1	Integrin subunit beta 1	Black	GSEA
PDGFC	Platelet derived growth factor C	Black	GSEA
SLIT3	Slit guidance ligand 3	antiquewhite4	GSEA
IL8(CXCL8)	C-X-C motif chemokine ligand 8	Steelblue	GSEA/CPDB
EDN1	Endothelin 1	Steelblue	CPDB

Genes related to angiogenesis and cell proliferation and migration (GO terms GO:0001525, GO:0090050, GO:0008543), from the white module were selected for further study since they are most related to the observed activities we are interested in. Six genes from the white module have been suggested as central genes (high and significant correlation to module average) (Table 4), and five of them, except SERPINE1, are only suggested by WGCNA, (i.e. are not differentially expressed compared to control in an enriched gene set). In total, 18 genes were selected for further study (Table 4). We can see that different methods did not suggest entirely overlapping genes, for instance, only 10 DEGs are highly and significantly correlated in the white module. Thus, combining DEG and WGCNA approaches enables to search potential genetic biomarkers which may be overlooked when using only one method.

### Gene Expression Analysis of Ginsenoside Rb2/Rg3 Combination Treatment Based on RNA-Seq and qPCR Data

**FIGURE 6:**
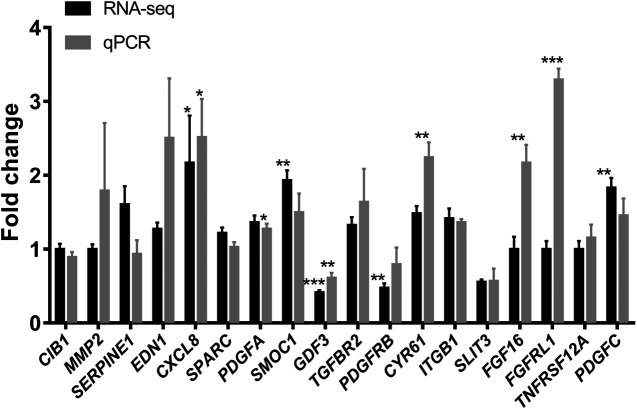
Elevation of mRNA expression in HUVECs treated with the combination of ginsenoside Rb2 (50 μM) and ginsenoside Rg3 (5 μM) or vehicle for 72 h as determined by real time qPCR (in gray) and compared to RNA-Seq data (in black). It can be seen that the Rb2/Rg3 combination upregulated several genes by 2- to 3-fold, such as *CXCL8, CYR61, FGF16* and *FGFRL1*. Data represents mean ± SD of four replicates. *p < 0.05, **p < 0.01, ***p < 0.001.

We next performed quantitative polymerase chain reaction (qPCR) on the 18 selected genes (Table 4) after the combination treatment of ginsenoside Rb2 (50 µM) and Rg3 (5 µM). The quantitative fold change between the control and the combination-treated cells of mRNA expression as well as the result of RNA-Seq raw counts of the 18 genes were presented in Figure 6. Notably, when compared with vehicle control, we can confirm that mRNA expression of *CXCL8, CYR61*, *FGF16 and FGFRL1* was highly up-regulated by the Rb2/Rg3 combination.

### The Ginsenoside Rb2/Rg3 Combination Acts *via* the CXCR1/2 CXCL8 (IL8)-Mediated PI3K/Akt and MAPK/ERK Signaling Pathways

**FIGURE 7:**
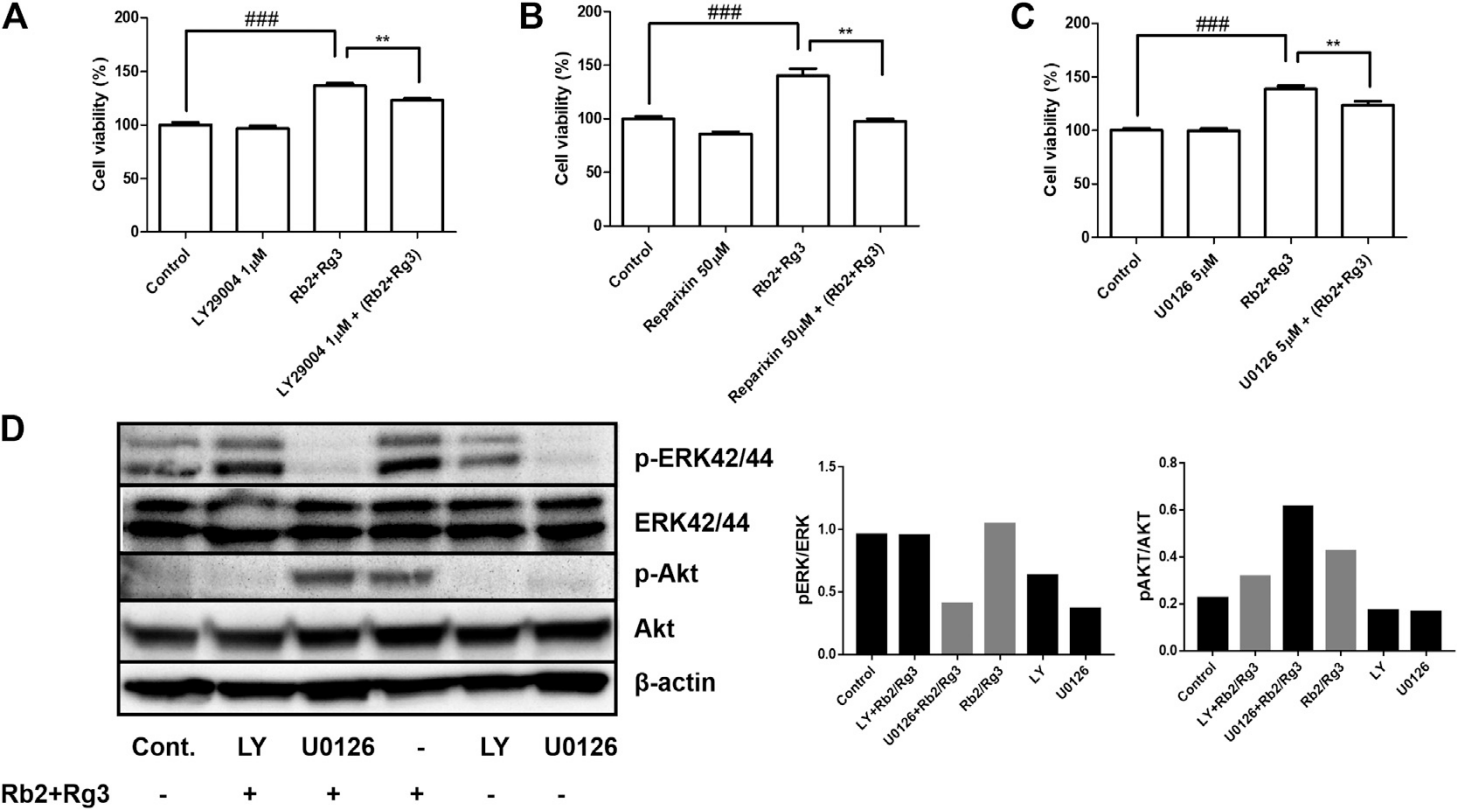
Effects of selective inhibitors of Akt (**(A)**; LY294002), CXCR1/2 (**(B)**; Reparixin) and ERK (**(C)**; U0126) on Rb2/Rg3-induced cell proliferation as monitored by the CCK-8 assay, indicating the pro-proliferative effects of the combination were due to activation of at least those three pathways. The effect of the CXCR1/2 inhibitor was most profound and entirely inhibited the stimulation of cell proliferation due to ginsenoside treatment. Data represents mean ± SD, n = 4. **p < 0.005 and ###*p* < 0.001. **(D)** Phosphorylation of Akt and ERK by the combination of Rb2 and Rg3 in the presence or absence of LY294002 and U0126 as measured by western blotting and the density of bands were measured. LY294002 and U0126 completely inhibited the promoting effects of Rb2 and Rg3 on the phosphorylation of Akt and ERK, respectively. These results demonstrate that Akt and ERK, and their phosphorylation, are essential for the pro-proliferative effects of the Rb2/Rg3 combination.

To further explore the molecular basis of the pro-proliferative effect of the ginsenoside Rb2/Rg3 combination in HUVECs, the CXCR1/2 antagonist Reparixin, the ERK inhibitor U0126 and the PI3K inhibitor LY294002 were used to block their respective signaling cascades. U0126, a kinase inhibitor of ERK to prevent phosphorylation of ERK, a family of MAPK, has been used in HUVEC at 10 µM in order to prove that their candidate drug induces angiogenesis via MAPK pathways (Wu et al., 2015). LY294002 blocks PI3K activity that phosphorylates Akt, resulting in unphosphorylated form of Akt. In this way we were able to test the hypothesis, based on mRNA analysis, that CYR61-stimulated IL-8 production leads to stimulation of the CXCR1/2 receptor, and thus the PI3K/Akt and MAPK/ERK signaling pathways are initiated consequently. The results of this experiment are shown in Figures 7A–C and demonstrate that the Rb2/Rg3-induced increase of HUVEC proliferation was significantly reduced in all three cases (and most pronouncedly by Reparixin), indicating that activation of CXCR1/2, MAPK/ERK and PI3K/Akt are involved in the mode of action of the combination. Western blot results (Figure 7D) demonstrated that Rb2/Rg3-induced Akt and ERK phosphorylation was significantly suppressed by LY294002 and U0126, while there was no reduction in the amount of total Akt and ERK protein present. This indicates that the activation of Akt and ERK *via* phosphorylation is also involved in the mode of action of the ginsenoside Rb2/Rg3 combination when stimulating HUVEC proliferation.

## Discussion

Shexiang Baoxin pill (SBP), which consists of seven materia medica, is widely used as a TCM formulation for the treatment of coronary heart disease. However, the modes of action of SBP in promoting angiogenesis have not yet been fully understood. In this regards, we previously modeled compound interactions of 22 constituent compounds detected in plasma (Zobir et al., submitted). However, while being able to predict synergies with a satisfactory performance and suggesting modes of action of compounds, this previous work did not biologically investigate mechanisms of synergistic interactions between compounds in promoting angiogenesis. Here, we focused on one particular combination of ginsenosides from SBP which has been identified as synergistic, that of ginsenosides Rb2 and Rg3, in order to investigate its molecular mode of action associated with angiogenesis.

Ginsenosides, isolated from *Panax ginseng*, are classified into two groups of saponins according to chemical structure: the dammarane-type having a 20(S)-protopanaxadiol/triol skeleton, and the oleanolic acid type possessing an oleanolic acid skeleton. Ginsenoside Rb2 and its hydrolytic product ginsenoside Rg3 belong to the protopanaxadiol type of dammarane ginsenosides. Ginsenosides Rb2 and Rg3 have multiple functions in immune regulation, anti-oxidation, anti-inflammation, anti-aging and anti-fatigue (Choi, 2002; Choi et al., 2015; Joo et al., 2015; Kim et al., 2016; Sun et al., 2017). However, their pro-angiogenic effects have not been vigorously studied, while their anti-angiogenesis along with anti-tumorigenic effects have been shown in several cancers (Mochizuki et al., 1995; Joo et al., 2015; Sun et al., 2016; Sun et al., 2017). As one of the few examples, related to pro-angiogenic effects, Kim et al. reported that ginsenoside Rg3 at 1, 5, 10 µM was able to prevent INS-1 cell death by increasing cell proliferation (Kim et al., 2016). Likewise, in our experimental conditions using HUVECs, ginsenoside Rg3 at lower concentrations (below 10 μM) markedly increased HUVEC proliferation. It is notable that most of the anticancer effects of ginsenoside Rg3 have been observed at much higher concentrations (>25 μM, although it varies on cell type). In addition, ginsenoside Rb2 has been reported that to enhance cell proliferation and migration by increasing EGF and EGFR levels (Choi, 2002). Given that ginsenosides have steroid-like structures, they are likely to bind to multiple steroid hormone receptors which may explain angiogenesis-modulating effects (Leung and Wong, 2010). Mechanisms of angiogenic activity of the saponin fraction has been proposed mainly through enhancement of tissue-type plasminogen activator (tPA) secretion together with suppression of plasminogen activator inhibitor-1 (PAI-1) secretion (Morisaki et al., 1995). Morisaki et al. reasoned that ginsenosides resembling sterols may contribute to the angiogenic activity of saponin (Morisaki et al., 1995). Even though the effect of these ginsenosides under different conditions (cell/tissue type, concentration, etc.) is yet to be understood in detail, it is evident that they target angiogenesis, and hence this work aimed to understand the mode of action of two synergistic components of a relevant traditional Chinese medicine, SBP, in this context.

While most of combination therapy aims at inhibiting angiogenesis in cancer treatments, combination pharmacotherapy can also provide benefits to prevent cardiovascular disease both in patients with high risk factors and individuals with existing cardiovascular disease (Working Group on the Summit on Combination Therapy for CVD et al., 2014). Our data demonstrate that a combination of ginsenosides Rb2 and Rg3 may act synergistically in promoting cellular proliferation as well as protecting endothelial cells from HCY-induced vascular damage. Ginsenoside Rb1, one of the protopanaxadiol type dammarane ginsenosides, has previously been shown to block HCY-induced endothelial dysfunction in porcine coronary arteries, suggesting potential clinical applications of ginsenosides in HCY-associated vascular injuries (Zhou et al., 2005). In our co-culture model of vascular tube formation, neither ginsenoside Rb2 alone nor ginsenoside Rg3 alone at the chosen concentrations was sufficient to rescue the tubes from HCY-induced damage; however, when combining these two ginsenosides together, the tube damage was largely reversed. The concentrations we used for the combination treatment were based on the Bliss synergy and the endothelial cell growth curves of each ginsenosides. Either ginsenoside Rb2 or ginsenoside Rg3 alone was able to show sigmoidal curves where the highest concentration hits plateau. Although synergism was observed throughout the areas of combination-treatments, ginsenoside Rb2 at 50 μM combined with ginsenoside Rg3 at 5 μM seem to be the best pair when compared to the single treatment.

In parallel with the potential therapeutic effects on reversing HCY-induced damage, the current study demonstrates that the ginsenoside Rb2/Rg3 combination can synergistically promote HUVEC proliferation. To elucidate the underlying molecular mechanism of the combination, RNA-Seq analysis of differentially expressed genes (DEGs) has been conducted in the present study. DEG analysis is a common method for identifying genes that play an important role in phenotypic effects, while transcription factor modifications such as reversible phosphorylation and missense mutations can be overlooked (Langfelder et al., 2011). Therefore, WGCNA together with DEG approaches have been applied in order to understand the mode of action of the compound combination further. A total of 377 Rb2/Rg3-induced DEGs were found to be highly enriched with angiogenesis- and extracellular matrix-related pathways. Moreover, the module with highest overlap of highly correlating DEGs and not conserved with control by WGCNA was associated with angiogenesis-related biological processes, however identifying somewhat distinct individual genes, indicating the importance of using different analysis methods in parallel. Since applications of RNA-Seq are not completely known yet as according to the huge data generated, researchers face challenges to analyze to avoid sequencing artifacts. Therefore, combining two different tools provides us more confident outcomes that those selected genes are less likely artifacts but produced by meaningful biological processes.

Thus, in this study, we have provided the first evidence that ginsenoside Rb2 and ginsenoside Rg3 synergistically promote endothelial cell proliferation and protect them against HCY-induced damage. RNA-Seq data by DEG and WGCNA analysis further indicate that angiogenesis pathways are transcriptionally perturbed by the combination of ginsenosides Rb2 and Rg3. Confirmed by qPCR, Western blotting, and pathway inhibition experiments, we hence conclude that the Rb2/Rg3 combination promotes endothelial cell proliferation through the CXCR1/2 CXCL8 (IL8)-mediated PI3K/Akt and MAPK/ERK signaling pathways (Heidemann et al., 2003) that may further effects on promoting angiogenesis.

More generally, the present study suggests a protocol on how to gain insight into the mode of action of a combination of two compounds from an originally more complex herbal formulation, which could be beneficial to standardize and simplify their complexity while maintaining efficacy.

## Data Availability

The original contributions presented in the study are publicly available. This data can be found here: https://www.ncbi.nlm.nih.gov/Traces/study/?acc=PRJNA671806.

## Abbreviations:

SBP, shexiang baoxin pill; HUVECs, human umbilical vein endothelial cells; DEGs, differentially expressed genes; WGCNA, weighted gene correlation network analysis; TCM, traditional Chinese medicine; CHD, coronary heart disease; eNOS, endothelial nitric oxide synthase; MAPK, mitogen-activated protein kinase; GSEA, gene set enrichment analysis; HDFs, human dermal fibroblasts; HCY, homocysteine
