# Supplementary material for: Combination of Ginsenosides Rb2 and Rg3 Promotes Angiogenic Phenotype of Human Endothelial Cells via PI3K/Akt and MAPK/ERK Pathways
Source: Front Pharmacol. 2021 Feb 10;12:618773. doi: 10.3389/fphar.2021.618773 (PMC7902932; doi:10.3389/fphar.2021.618773)
Supplement: Supplementary file 1 [file datasheet1.docx]

**Supplementary Material**


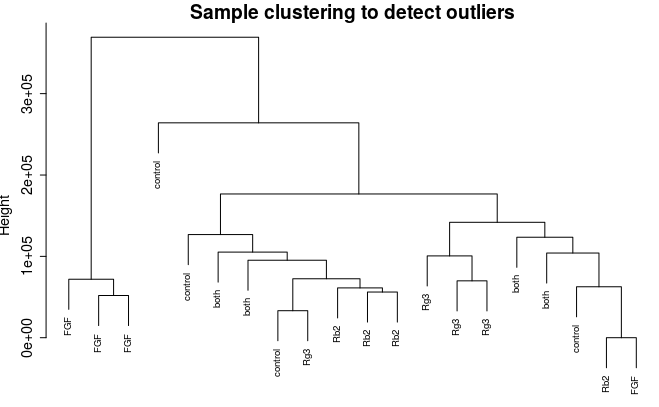


**Figure S1** Hierarchical clustering using Euclidean distance in gene expression space. The distinction of FGF from Rb2, Rg3 and the combination of Rb2 and Rg3 (both) indicates differing gene expression profiles, and so it is excluded from further study with WGCNA


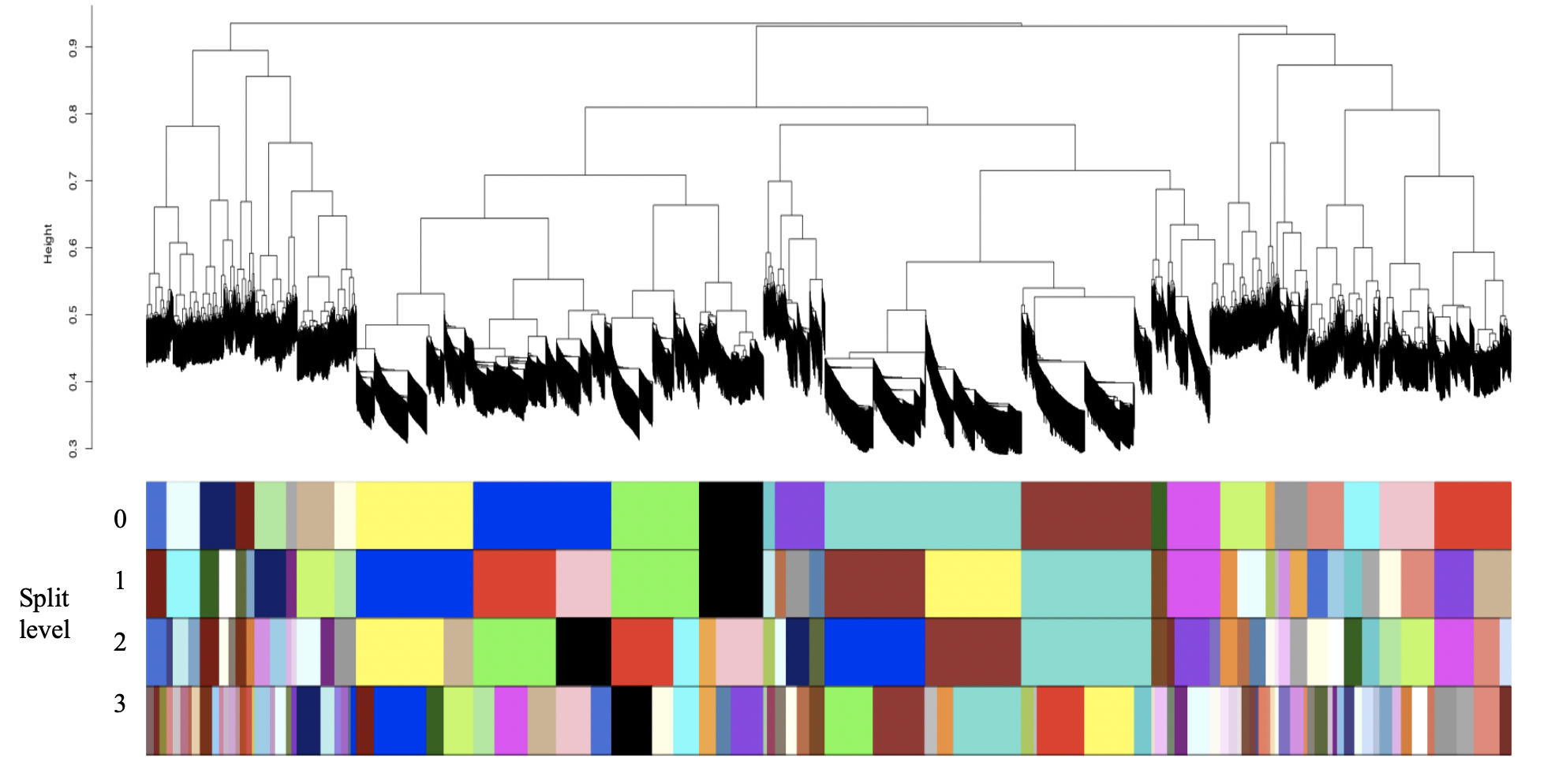


**Figure S2** Hierarchical clustering of modules. Networks were created for each condition and modules formed at four levels, with each level of representing cutting the tree at different heights. The lowest level (split level = 3) contains modules whose genes have the highest correlation to each other, modules are smallest. The clustering of the genes is clearly differentiated. Network clustering resulted in 93 distinct modules and they were perturbation tested against the control which gave the resultant Z summary score. 15 modules showed strong evidence of conservation with the control cases, and are assumed to represent the underlying biology of the cell.

**Table S1** Network permutation results (Zsummary, median rank, and gene-module correlation (defined as cor>0.7, p <0.01)), differentially expressed genes (DEGs) and the overlap between the two. This is used to direct module importance and gene selection

| Module  Name | Module  Size | Zsummary | | Median  rank | # DEGs | # highly correlated genes | # highly correlated genes that are DEGs |
| --- | --- | --- | --- | --- | --- | --- | --- |
| skyblue | 136 | -0.87 | 85 | | 2 | 3 | #N/A |
| honeydew | 35 | -0.87 | 90 | | 2 | #N/A | #N/A |
| plum2 | 78 | -0.80 | 72 | | 5 | #N/A | #N/A |
| mediumpurple2 | 57 | -0.70 | 80 | | 1 | 45 | 1 |
| yellow3 | 22 | -0.65 | 92 | | #N/A | #N/A | #N/A |
| violet | 118 | -0.49 | 87 | | 2 | 45 | 1 |
| brown2 | 48 | -0.41 | 83 | | 1 | #N/A | #N/A |
| brown4 | 94 | -0.14 | 72 | | 1 | #N/A | #N/A |
| darkorange2 | 94 | -0.12 | 78 | | 8 | #N/A | #N/A |
| navajowhite1 | 39 | 0.17 | 79 | | 2 | 19 | 1 |
| blue2 | 46 | 0.19 | 69 | | #N/A | #N/A | #N/A |
| coral2 | 62 | 0.25 | 85 | | 3 | #N/A | #N/A |
| lightcyan1 | 96 | 0.41 | 62 | | #N/A | #N/A | #N/A |
| coral | 32 | 0.45 | 78 | | 2 | #N/A | #N/A |
| yellow4 | 61 | 0.64 | 47 | | 1 | #N/A | #N/A |
| firebrick4 | 49 | 0.66 | 60 | | #N/A | 42 | #N/A |
| mediumorchid | 62 | 0.84 | 50 | | 1 | 46 | 1 |
| lavenderblush3 | 67 | 1.01 | 51 | | #N/A | 55 | #N/A |
| plum3 | 44 | 1.07 | 55 | | 2 | #N/A | #N/A |
| lightcoral | 54 | 1.12 | 20 | | 1 | 48 | #N/A |
| mediumpurple4 | 25 | 1.12 | 68 | | 1 | 19 | 1 |
| magenta4 | 38 | 1.23 | 72 | | 2 | #N/A | #N/A |
| palevioletred3 | 71 | 1.37 | 63 | | 6 | 53 | 5 |
| darkslateblue | 92 | 1.42 | 73 | | 4 | 41 | 1 |
| darkolivegreen4 | 49 | 1.46 | 57 | | 2 | #N/A | #N/A |
| salmon | 228 | 1.54 | 35 | | 7 | #N/A | #N/A |
| skyblue3 | 110 | 1.71 | 70 | | 7 | #N/A | #N/A |
| darkgrey | 149 | 1.82 | 46 | | 8 | 92 | 6 |
| salmon2 | 41 | 1.87 | 85 | | 3 | #N/A | #N/A |
| mediumpurple3 | 99 | 1.88 | 84 | | 6 | 37 | 1 |
| thistle | 41 | 1.93 | 47 | | 1 | #N/A | #N/A |
| darkseagreen3 | 34 | 2.09 | 81 | | #N/A | 20 | #N/A |
| skyblue4 | 24 | 2.10 | 42 | | #N/A | #N/A | #N/A |
| maroon | 70 | 2.11 | 87 | | 2 | 30 | #N/A |
| coral1 | 63 | 2.12 | 75 | | 2 | #N/A | #N/A |
| antiquewhite4 | 62 | 2.17 | 49 | | 4 | 52 | 2 |
| white | 136 | 2.18 | 45 | | 11 | 94 | 10 |
| darkviolet | 46 | 2.23 | 71 | | #N/A | 31 | #N/A |
| lightsteelblue1 | 98 | 2.30 | 73 | | 3 | 3 | #N/A |
| grey60 | 192 | 2.37 | 31 | | 4 | #N/A | #N/A |
| lightpink4 | 69 | 2.39 | 33 | | 2 | 53 | 2 |
| paleturquoise | 122 | 2.42 | 55 | | 1 | 80 | 1 |
| orangered4 | 106 | 2.61 | 42 | | 2 | 57 | #N/A |
| orangered3 | 58 | 2.62 | 56 | | #N/A | 33 | #N/A |
| skyblue1 | 61 | 2.64 | 30 | | #N/A | #N/A | #N/A |
| orange | 148 | 2.85 | 68 | | 10 | 72 | 5 |
| indianred4 | 52 | 2.89 | 77 | | 4 | 28 | 2 |
| midnightblue | 206 | 3.08 | 54 | | 4 | 116 | #N/A |
| thistle3 | 43 | 3.11 | 58 | | 1 | #N/A | #N/A |
| lightpink3 | 38 | 3.23 | 60 | | #N/A | 22 | #N/A |
| coral3 | 29 | 3.25 | 48 | | 1 | #N/A | #N/A |
| navajowhite2 | 71 | 3.43 | 28 | | 2 | 53 | 1 |
| antiquewhite2 | 32 | 3.53 | 32 | | 1 | 26 | 1 |
| palevioletred2 | 39 | 3.63 | 24 | | 1 | 33 | 1 |
| gold | 100 | 3.94 | 62 | | #N/A | #N/A | #N/A |
| skyblue2 | 62 | 4.19 | 59 | | 2 | #N/A | #N/A |
| darkolivegreen | 117 | 4.30 | 50 | | 3 | #N/A | #N/A |
| lightsteelblue | 55 | 4.35 | 82 | | 2 | #N/A | #N/A |
| lavenderblush2 | 36 | 4.69 | 21 | | 1 | 28 | 1 |
| royalblue | 177 | 4.98 | 22 | | 6 | 114 | 5 |
| plum | 61 | 5.31 | 28 | | #N/A | #N/A | #N/A |
| purple | 281 | 5.59 | 19 | | 14 | 180 | 9 |
| salmon4 | 72 | 5.63 | 60 | | 2 | #N/A | #N/A |
| thistle2 | 75 | 5.69 | 31 | | 2 | 3 | #N/A |
| honeydew1 | 66 | 5.94 | 63 | | 2 | #N/A | #N/A |
| bisque4 | 93 | 6.22 | 48 | | 1 | 34 | #N/A |
| thistle1 | 73 | 7.14 | 57 | | 2 | #N/A | #N/A |
| darkseagreen4 | 64 | 7.26 | 34 | | 2 | 25 | #N/A |
| ivory | 95 | 7.40 | 53 | | 2 | 50 | 1 |
| floralwhite | 95 | 7.70 | 71 | | 4 | 38 | 1 |
| sienna3 | 111 | 7.74 | 47 | | 5 | 55 | 2 |
| tan | 248 | 7.95 | 12 | | 6 | 183 | 4 |
| steelblue | 132 | 8.00 | 15 | | 6 | 106 | 5 |
| darkgreen | 154 | 8.05 | 16 | | 5 | 112 | 4 |
| lightyellow | 186 | 8.11 | 27 | | 12 | 122 | 8 |
| plum1 | 108 | 8.21 | 58 | | #N/A | #N/A | #N/A |
| yellowgreen | 111 | 8.24 | 29 | | 4 | 24 | #N/A |
| grey | 134 | 8.87 | 39 | | 3 | #N/A | #N/A |
| darkmagenta | 112 | 8.95 | 23 | | 7 | 77 | 6 |
| darkturquoise | 151 | 9.83 | 16 | | 6 | 90 | 4 |
| lightcyan | 193 | 9.91 | 34 | | 4 | #N/A | #N/A |
| saddlebrown | 133 | 10.07 | 56 | | 1 | 41 | 1 |
| lightgreen | 187 | 10.74 | 3 | | 6 | 168 | 6 |
| darkorange | 141 | 11.12 | 11 | | #N/A | #N/A | #N/A |
| darkred | 158 | 11.94 | 4 | | 2 | 140 | 2 |
| pink | 306 | 12.01 | 15 | | 10 | 211 | 8 |
| greenyellow | 258 | 12.06 | 18 | | 15 | 152 | 8 |
| cyan | 223 | 12.38 | 24 | | 8 | 119 | 6 |
| magenta | 287 | 13.32 | 10 | | 8 | #N/A | #N/A |
| black | 357 | 15.09 | 10 | | 20 | 257 | 14 |
| green | 422 | 19.04 | 9 | | 9 | 226 | 5 |
| yellow | 434 | 19.12 | 5 | | 7 | #N/A | #N/A |
| red | 415 | 19.97 | 6 | | 11 | 254 | 7 |
| blue | 454 | 20.67 | 2 | | 13 | #N/A | #N/A |
| brown | 451 | 21.26 | 2 | | 13 | #N/A | #N/A |
| turquoise | 594 | 24.35 | 6 | | 13 | 369 | 6 |

Table S2. Primer sequence used in this study

| Primer name | Sequence (from 5’ to 3’) |
| --- | --- |
| CIB1  Forward  Reverse | CACGTCATCTCCCGTTC  CTGCTGTCACAGGACAAT |
| MMP2  Forward  Reverse | GAGAACCAAAGTCTGAAGAG  GGAGTGAGAATGCTGATTAG |
| SERPINE1  Forward  Reverse | ATCCACAGCTGTCATAGTC  CACTTGGCCCATGAAAAG |
| TNFRSF12A  Forward  Reverse | GAGAGAGAAGTTCACCACC  CATTGTCACTGGATCAGC |
| FGF16  Forward  Reverse | TCGGAATCCTGGAGTTTATC  AGTTTCTTCGACCCATAGAG |
| FGFRL1  Forward  Reverse | ATCACGTGGATGAAGGAC  TGGATCACATCCACCTTG |
| SPARC  Forward  Reverse | ATCTAAATCCACTCCTTCCACAG  CACCGTTAATGTATTCACTTAAATC |
| PDGFA  Forward  Reverse | GCTTTATTGCCAGTGTGCGGTCTT  TGCACTGTCTCTTTGTTCTCCCGA |
| SMOC1  Forward  Reverse | TAACAAGCGGGAGATGAA  CACAGTAGTCGGTGAAACG |
| GDF3  Forward  Reverse | CCAAGGTTTCTTTCTTTACCC  AATGTCAACTGTTCCCTTTC |
| TGFBR2  Forward  Reverse | CGTGAAGAACGACCTAACC  CCACCTGCCCACTGTTAG |
| PDGFRB  Forward  Reverse | GGGAAGAGAAGTTTGAGATTC  TTCTTTTTGTAACCTTCGCC |
| CYR61  Forward  Reverse | CGAGGTGGAGTTGACGAGAA  GCACTCAGGGTTGTCATTGGT |
| ITGB1  Forward  Reverse | ATTCCCTTTCCTCAGAAGTC  TTTTCTTCCATTTTCCCCTG |
| PDGFC  Forward  Reverse | TTGGAAGAAAATCCAGAGTG  ATTGCAATTGTGGAGACAAC |
| SLIT3  Forward  Reverse | CAATGGCTACACATGCACCT  TGATGAGCTTCTCGCATCTG |
| IL8(CXCL8)  Forward  Reverse | ATGACTTCCAAGCTGGCCGTGGCT  TCTCAGCCCTCTTCAAAAACTTCTC |
| EDN1  Forward  Reverse | CAGCGTCCTCGTTCAAAACATT CCCCAGATGAAAGAAGAGACCA |
| GAPDH  Forward  Reverse | TGAACGGGAAGCTCACTGG  TCCACCACCCTGTTGCTGTA |
